# Pulmonary function and risk factors in pediatric chest tightness variant asthma: a case-control study

**DOI:** 10.3389/fped.2026.1842381

**Published:** 2026-08-11

**Authors:** Ruo-Yu Cao, Wei-Chao He, Ya-Bin Yu, Shan-Shan Tong

**Affiliations:** 1 Department of Pediatrics, Cangzhou Central Hospital, Cangzhou, Hebei, China; 2 Department of Pediatrics, Hejian City Hospital of TCM, Cangzhou Central Hospital Medical Group, Hejian City, Hebei, China

**Keywords:** chest tightness variant asthma, children, fractional exhaled nitric oxide (FENO), pulmonary function, risk factors

## Abstract

**Objective:**

To investigate the pulmonary function characteristics and risk factors for pediatric chest tightness variant asthma (CTVA).

**Methods:**

A retrospective case-control study was conducted. Clinical data of 39 children diagnosed with CTVA in our outpatient department from February 2022 to February 2024 were analyzed. Fifty healthy children undergoing physical examinations during the same period were enrolled as controls. All participants underwent fractional exhaled nitric oxide (FeNO) measurement and pulmonary ventilation function tests at initial diagnosis or physical examination. FeNO levels and pulmonary function parameters were compared between groups. Baseline demographic and clinical data were recorded. Univariate logistic regression analyses were performed to explore factors associated with pediatric CTVA. Due to the limited sample size (39 cases) and an event-per-variable ratio below the recommended minimum of 10:1 for reliable multivariable modeling, multivariate analysis was not performed.

**Results:**

FeNO levels were significantly higher in the CTVA group than in the healthy control group (*p* = 0.016). FEV1/FVC, PEF, FEF25, FEF50, and FEF75 were significantly lower in the CTVA group (all *p* < 0.05), whereas FEV1, FVC, and MMEF showed no significant differences (*p* > 0.05). Significant intergroup differences were observed for obesity, inhalant allergen sensitization, allergic rhinitis, atopic dermatitis, history of recurrent respiratory infections, and family history of allergic diseases (all *p* < 0.05). Univariate logistic regression analysis confirmed these six factors as significantly associated with pediatric CTVA (all *p* < 0.05).ntified these six factors as potential independent risk factors for pediatric CTVA (all *p* < 0.05)

**Conclusion:**

Children with CTVA have higher FeNO levels and lower FEV1/FVC, PEF, FEF25, FEF50, and FEF75 compared to healthy peers. Obesity, inhalant allergens, allergic rhinitis, atopic dermatitis, recurrent respiratory infections, and family history of allergic diseases showed significant univariate associations with pediatric CTVA in this exploratory cohort. Combined assessment of FeNO and small airway parameters may aid in the diagnostic evaluation of CTVA, although further diagnostic accuracy studies are needed.

## 1 Introduction

Asthma (classic asthma, CA) is a heterogeneous disease with variable symptoms. Some patients present only with a cough, or have a cough as the main symptom, while others experience recurrent chest tightness as the sole clinical symptom, without typical asthma manifestations such as recurrent wheezing and shortness of breath (1, 2). The pathogenesis of these atypical forms is similar to that of classic asthma, with objective evidence of variable airflow limitation and effective response to anti-asthma treatment. These conditions have been termed cough variant asthma (CVA) (3) and chest tightness variant asthma (CTVA), representing distinct clinical subtypes (4).

Chest tightness variant asthma (CTVA) is characterized by chest tightness or frequent sighing as the sole or main symptom, without wheezing, shortness of breath, or other typical asthma manifestations, and with no wheezing sounds on chest auscultation (5). CTVA patients exhibit airway hyperresponsiveness and reversible airflow limitation—pathophysiological features typical of asthma—and respond effectively to inhaled corticosteroids (ICS) or ICS combined with long-acting beta2-agonists (LABA) (6, 7). In recent years, with ecological and lifestyle changes and industrialization, the incidence of pediatric CTVA has been increasing annually. Due to the lack of typical symptoms, pediatric CTVA is easily misdiagnosed or overlooked, preventing affected children from receiving standard treatment. As the disease progresses, it may develop into classic asthma, affecting growth and development (8).

Research indicates that CTVA is influenced by various factors, including the child's own developmental factors, environmental factors, and genetic factors. This retrospective case-control study aimed to explore potential associated factors for pediatric CTVA using questionnaires and statistical analysis. The results are reported as follows.

## 2 Study subjects and methods

### 2.1 Ethical statement

This study was approved by the Ethics Committee of Cangzhou Central Hospital (approval number: TR2020403 Written informed consent was obtained from the parents or legal guardians of all participants. All procedures were conducted in accordance with the Declaration of Helsinki.

### 2.2 Study subjects

A retrospective analysis was conducted on the clinical data of 39 children diagnosed with CTVA in our outpatient department from February 2022 to February 2024. Additionally, 50 healthy children undergoing physical examinations during the same period were selected as a control group. Age and sex were matched between groups (no significant differences, *p* > 0.05).

Inclusion criteria for the CTVA group: (1) recurrent chest tightness as the main or sole clinical symptom, without wheezing on lung examination; (2) no significant abnormalities on chest x-ray or CT scan; (3) no history of chronic cough or wheezing; (4) exclusion of chest tightness caused by other systemic diseases; (5) age 3–12 years, with a positive bronchial provocation test (see Section 2.3.3 for details); (6) significant symptom improvement after initial treatment with inhaled corticosteroids combined with long-acting beta2-agonists.

Exclusion criteria for CTVA group: (1) presence of other respiratory diseases such as pulmonary infection or bronchiectasis; (2) significant organ dysfunction; presence of mental system diseases or consciousness disorders; (3) presence of other severe systemic diseases such as autoimmune diseases, myocardial infarction, heart failure, malignant tumors; (4) previously diagnosed with bronchopulmonary dysplasia.

Inclusion criteria for healthy controls: (1) no history of severe cardiopulmonary or other systemic diseases, especially no history of asthma, tuberculosis, pleurisy, or recurrent bronchitis; (2) no history of upper or lower respiratory tract infections in the past 4 weeks; (3) never smoked and no passive smoking exposure; (4) no history of harmful gas or dust exposure; (5) normal chest development, height and weight within normal range. All healthy controls had no clinical evidence of allergic diseases or airway hyperresponsiveness.

Exclusion criteria for healthy controls: (1) children with epilepsy currently undergoing medication; (2) currently using beta-receptor blockers for any reason (including eye drops); (3) resting heart rate >120 beats per minute. Additionally, subjects who did not cooperate well during testing, failed to meet quality control standards, or were found to have rhinitis or upper respiratory tract infections during measurement were excluded.

### 2.3 Research methods

#### 2.3.1 FeNO measurement

FeNO measurement was performed prior to pulmonary function and airway responsiveness testing using the NIOX mobile FeNO measurement system (Nox Company, Sweden). A single-breath exhalation method was used for real-time measurement, following the American Thoracic Society (ATS)/European Respiratory Society (ERS) 2005 recommendations for children (9). Quality control: measurements were performed with exhalation pressure of 10–20 cmH₂O for 6 s for children aged 3–6 years and 10 s for those aged >6 years, with plateau phase of at least 2 s. Acceptable maneuvers required three reproducible readings within 10% or 5 ppb of each other, and the mean value was recorded.

#### 2.3.2 Pulmonary function tests

All subjects underwent pulmonary function tests using a calibrated spirometer (MasterScreen, CareFusion, Germany), including assessments of pulmonary ventilation [forced vital capacity (FVC), forced expiratory volume in 1 s (FEV1), FEV1/FVC ratio, peak expiratory flow (PEF)], small airway function [maximal expiratory flow at 75% of FVC (MEF75), MEF50, MEF25, also denoted as FEF75, FEF50, FEF25 respectively], and maximal mid-expiratory flow (MMEF). Quality control: all tests were performed according to ATS/ERS 2019 standards (10). At least three acceptable maneuvers were obtained, with reproducibility criteria of FEV1 and FVC within 0.15 L. Results were expressed as percentage of predicted values using reference equations for Chinese children.

#### 2.3.3 Bronchial provocation test

Bronchial provocation testing was performed using the histamine challenge method according to the standardized protocol. Histamine diphosphate (Sigma-Aldrich, USA) was diluted with normal saline to produce doubling concentrations from 0.03 mg/mL to 16 mg/mL. The test was initiated with inhalation of normal saline, followed by increasing concentrations of histamine using a nebulizer (DeVilbiss 646, USA) and tidal breathing for 2 min at each concentration. FEV1 was measured 30 s after each inhalation. The test was terminated when FEV1 dropped by ≥20% from baseline (positive) or the maximum concentration was reached. The provocative concentration causing a 20% fall in FEV1 (PC20) was calculated. PC20 ≤ 8 mg/mL was defined as positive for airway hyperresponsiveness.

#### 2.3.4 Diagnostic criteria for obesity and allergen sensitization

Obesity: defined as body mass index (BMI) ≥ 95th percentile for age and sex according to the Chinese growth charts for children aged 3–12 years (11).

Inhalant allergen sensitization: defined as a positive skin prick test (SPT) or serum specific IgE (≥0.35 kU/L) to at least one common inhalant allergen (including house dust mite, pollen, mold, animal dander). SPT was performed using standardized allergen extracts (ALK-Abelló, Denmark); a wheal diameter ≥3 mm larger than negative control was considered positive.

#### 2.3.5 Statistical record of baseline data

The following baseline data were recorded for each participant: basic information including gender, age, and obesity status (yes/no); maternal pregnancy conditions such as history of medication use, pregnancy-related diseases, and radiation exposure; birth conditions including mode of delivery (vaginal delivery or cesarean section); feeding methods (breastfeeding, mixed feeding, or formula feeding); allergen profiles (inhalant allergens and food allergens); medical history comprising allergic rhinitis, atopic dermatitis, recurrent respiratory infections, and anorexia; living environment factors including smoking exposure, presence of indoor plants, and pet ownership; and family history of allergic diseases in parents or first-degree relatives (e.g., asthma, allergic rhinitis, eczema, urticaria, angioneurotic edema, and drug allergies).

### 2.4 Statistical analysis

Statistical analysis was performed using SPSS version 26.0 (IBM Corp., Armonk, NY, USA). Continuous variables with normal distribution were expressed as mean ± standard deviation and compared using independent t-test. Non-normally distributed variables were expressed as median (interquartile range) and compared using Mann–Whitney U test. Categorical variables were compared using chi-square test. Given the limited sample size (39 cases) and an event-per-variable ratio of approximately 6.5:1 (below the recommended minimum of 10:1 for reliable multivariable modeling), multivariate logistic regression was not performed to avoid model overfitting (12, 13). Instead, univariate logistic regression was applied to each of the six variables separately to explore factors associated with CTVA. All significant factors from univariate analysis are reported with odds ratios and 95% confidence intervals. Results should be interpreted as exploratory and hypothesis-generating. A two-sided *p* < 0.05 was considered statistically significant. No sample size calculation was performed *a priori* due to the retrospective design; this is acknowledged as a limitation.

## 3 Results

### 3.1 Comparison of general conditions between the two groups

As shown in Table 1, the CTVA group included 39 children (23 males, 16 females; mean age 9.62 ± 2.36 years). The healthy control group included 50 children (30 males, 20 females; mean age 9.33 ± 2.48 years). Age and sex were well matched between groups (*p* > 0.05).

**Table 1:** Demographic characteristics of the study groups.

Group	*n*	Male	Female	Average age (Years)
CTVA group	39	23	16	9.62 ± 2.36
Healthy control	50	30	20	9.33 ± 2.48
t/x^2^				0.559
p				0.578

### 3.2 Comparison of FeNO values between the two groups

As shown in Table 2, FeNO levels were significantly higher in the CTVA group than in the healthy control group (median 14.16 vs. 9.09 ppb, *p* = 0.016).

**Table 2:** Comparison of FeNO levels between groups.

Group	*n*	Median FeNO (ppb) (IQR)
CTVA group	39	14.16 (8.02, 24.05)
Healthy Control Group	50	9.09 (7.07, 18.58)
Z		3.108
p		0.016

### 3.3 Comparison of pulmonary function between the two groups

As shown in Table 3, FEV1/FVC, PEF, FEF25, FEF50, and FEF75 were significantly lower in the CTVA group compared to healthy controls (all *p* < 0.05). No significant differences were observed in FEV1, FVC, or MMEF (*p* > 0.05).

**Table 3:** Comparison of pulmonary function parameters between groups.

Parameter	CTVA group (*n* = 39)	Healthy control (*n* = 50)	Statistic	*p*
FEV1 (%)	99.15 ± 11.81	102.72 ± 6.86	t = 4.797	0.084
FVC [M(P25-P75)]	99.31 (89.27–105.29)	95.40 (90.17–99.66)	Z = 1.818	0.119
FEV1/FVC [M(P25-P75)]	0.999 (0.968–1.080)	1.081 (1.040–1.104)	Z = 3.506	0.001
PEF (%)	94.42 ± 12.92	106.39 ± 14.15	t = 4.112	0.001
MMEF (%)	79.53 ± 21.32	87.77 ± 18.99	t = 1.924	0.057
FEF25 [M(P25-P75)]	87.81 (81.44–102.21)	104.73 (92.07–112.70)	Z = 4.586	0.002
FEF50 [M(P25-P75)]	79.16 (64.79–93.76)	86.18 (79.06–97.68)	Z = 1.750	0.035
FEF75 [M(P25-P75)]	66.51 (53.11–95.91)	72.30 (66.15–81.91)	Z = 1.791	0.036

### 3.4 Univariate analysis of factors associated with pediatric CTVA

As shown in Table 4, univariate logistic regression analysis revealed that obesity, inhalant allergens, allergic rhinitis, atopic dermatitis, history of recurrent respiratory infections, and family history of allergic diseases were significantly associated with pediatric CTVA (all *p* < 0.05). Due to the limited sample size (*n* = 39 cases) and an event-per-variable ratio of approximately 6.5:1 (below the recommended 10:1 threshold for reliable multivariable modeling), multivariable adjustment was not performed to avoid model overfitting. Therefore, all associations should be interpreted as exploratory and hypothesis-generating, and the odds ratios presented are derived from univariate models without adjustment for potential confounders. The distribution of each variable between the two groups is also presented in Table 4.

**Table 4:** Univariate analysis of factors associated with pediatric CTVA.

Variable	CTVA group (*n* = 39)	Healthy control (*n* = 50)	OR	95% CI	*p*
Gender (Male/Female)	23/16	30/20	1.042	0.457–2.376	0.922
Age (years)	9.62 ± 2.36	9.33 ± 2.48	1.048	0.887–1.239	0.578
Obesity (present)	21	14	3.267	1.236–12.547	0.014
Maternal medication history	2	2	1.286	0.173–9.548	0.794
Delivery (cesarean)	24	33	0.764	0.321–1.819	0.663
Inhalant allergens	24	11	2.179	1.173–11.338	0.031
Food allergens	6	6	1.306	0.389–4.386	0.643
Allergic rhinitis	15	5	2.875	1.127–7.328	0.029
Atopic dermatitis	12	5	3.605	1.036–12.537	0.046
Recurrent respiratory infections	15	8	1.945	0.861–4.448	0.015
Anorexia	11	15	0.927	0.360–2.386	0.854
Smoking environment	11	15	0.932	0.362–2.399	0.762
Indoor plants	17	20	—	—	—
Pets	11	15	—	—	—
Family history of allergic diseases	14	5	4.816	2.092–11.045	0.002

OR values are derived from univariate logistic regression. Multivariable adjustment was not performed due to small sample size (EPV < 10:1). Results are exploratory and require validation in larger cohorts.

## 4 Discussion

This case-control study investigated pulmonary function characteristics and factors associated with pediatric chest tightness variant asthma (CTVA). The key findings are: (1) Children with CTVA have significantly higher FeNO levels and lower small airway function parameters (FEF25, FEF50, FEF75) and FEV1/FVC, PEF compared to healthy controls, while FEV1 and FVC are preserved; (2) Obesity, inhalant allergen sensitization, allergic rhinitis, atopic dermatitis, recurrent respiratory infections, and family history of allergic diseases showed significant associations with pediatric CTVA in univariate analysis; (3) Passive smoking exposure was not identified as a significant factor in this cohort, likely due to limited sample size and selection criteria.

Our findings are consistent with several recent studies on pediatric CTVA. Zhou et al. (14) reported that CTVA children exhibit preserved large airway function but significant small airway dysfunction compared to healthy controls. Zhang et al. (15) found that FEV1% in CTVA children (103.3%) was higher than in classic asthma (94.9%) and cough variant asthma (94.2%), while the proportion of severe small airway decline in CTVA (14%, 6/42) was similar to CVA (18%, 3/17) but lower than in classic asthma (37%, 18/48). Feng et al. (5) demonstrated that combined exercise challenge testing and FeNO improved diagnostic accuracy for CTVA in children. Our results corroborate that small airway dysfunction is a prominent feature of CTVA, and FeNO elevation, though less pronounced than in classic asthma, is detectable.

The observation that FEF25, FEF50, and FEF75—markers of small airway function—were significantly reduced in CTVA children, whereas FEV1 and FVC remained normal, suggests that small airway impairment may be an early pathophysiological event in CTVA. Yan et al. (7) reported that chest tightness sensation can occur early during acetylcholine-induced bronchoconstriction, often preceding significant FEV1 decline. Huang et al. (16) observed that asthmatic patients with chest tightness had more pronounced small airway dysfunction than those without. These observations support the hypothesis that subtle changes in small airway caliber are detectable by patients and manifest as chest tightness, even when large airway function remains within normal limits. Therefore, assessment of small airway parameters (FEF25–75) should be incorporated into the routine pulmonary function evaluation of children presenting with unexplained chest tightness.

Turning to fractional exhaled nitric oxide (FeNO), our CTVA group had significantly higher FeNO levels than healthy controls (median 14.16 vs. 9.09 ppb, *p* = 0.016). Nguyen et al. (17) performed bronchial mucosal biopsies in six CTVA patients and found eosinophilic infiltration, basement membrane thickening, and epithelial inflammation-pathological hallmarks of asthma. The FeNO levels in our CTVA children were elevated but generally lower than reported values for classic childhood asthma (18), consistent with the notion that CTVA represents a milder or distinct inflammatory phenotype. The diagnostic value of combining FeNO with small airway indicators has been suggested in previous studies (19). However, our study did not perform a formal diagnostic accuracy analysis (e.g., ROC curve) to derive sensitivity, specificity, or optimal cut-off values for combined testing. This is a limitation, and future prospective studies with larger samples and predefined diagnostic thresholds are needed to validate the clinical utility of FeNO–small airway parameter combinations in CTVA diagnosis.

With respect to associated factors, our univariate analysis identified obesity, inhalant allergens, allergic rhinitis, atopic dermatitis, recurrent respiratory infections, and family history of allergic diseases as factors significantly associated with CTVA in this exploratory cohort. These findings align with the known epidemiology of asthma and atopy. Obesity may contribute via mechanical compression of airways and systemic inflammation (20). Inhalant allergens and atopic comorbidities (allergic rhinitis, atopic dermatitis) reflect a type-2 inflammatory endotype, which is consistent with elevated FeNO (21). Recurrent respiratory infections in early childhood can disrupt airway epithelial integrity, promote viral-induced inflammation, and potentially increase susceptibility to allergic sensitization (22). Family history of allergic diseases is a well-established risk factor for all asthma phenotypes.

Interestingly, passive smoking was not identified as a significant factor in our study, in contrast to some previous reports. However, this finding should be interpreted with caution because the control group was specifically selected to have no passive smoking exposure (inclusion criterion 3), which effectively eliminated between-group variability for this variable. Additionally, the small sample size (*n* = 39 cases) may have limited statistical power to detect modest effects. Therefore, the absence of a significant association for passive smoking in this study does not exclude its potential role, and larger, multi-center studies with more diverse exposure levels are needed to reliably assess this factor.

### 4.1 Limitations

This study has several limitations. First, the retrospective, single-center design with a small sample size (39 cases, 50 controls) limits generalizability. No *a priori* sample size calculation was performed. Second, we did not include a classic asthma control group; therefore, we cannot distinguish which functional or risk factor patterns are unique to CTVA vs. shared with typical asthma. Third, due to the limited sample size (39 cases) and an event-per-variable ratio below the recommended minimum of 10:1 for reliable multivariable modeling, we did not perform multivariable adjustment. Consequently, the odds ratios presented are derived from univariate models and may be subject to confounding, and all associations should be interpreted as exploratory rather than confirmatory. Fourth, we did not perform a formal diagnostic accuracy analysis (e.g., ROC curve) for combined FeNO and small airway parameters; therefore, any suggestion of diagnostic value remains preliminary and requires prospective validation. Fifth, the bronchial provocation test protocol (histamine) and cut-off values (PC20 ≤ 8 mg/mL) follow local guidelines but may not be directly comparable to studies using methacholine. Sixth, the absence of passive smoking as a risk factor may be due to type II error or selection bias.

### 4.2 Future directions

Future research should include multi-center, prospective cohorts with adequate sample sizes, incorporate a classic asthma control group, validate the diagnostic performance of FeNO–small airway parameter combinations, and explore underlying mechanisms linking obesity and recurrent infections to CTVA pathogenesis.

## 5 Conclusion

In summary, children with chest tightness variant asthma have higher FeNO levels and lower small airway function parameters (FEF25, FEF50, FEF75) and FEV1/FVC, PEF compared to healthy children. Obesity, inhalant allergen sensitization, allergic rhinitis, atopic dermatitis, recurrent respiratory infections, and a family history of allergic diseases showed significant associations with pediatric CTVA in this exploratory cohort. The combination of FeNO and small airway indicators may warrant further investigation in the diagnostic evaluation of CTVA, but formal diagnostic accuracy studies are needed. Given the exploratory nature of this study and its small sample size, all findings require validation in larger, prospective, multi-center cohorts with inclusion of classic asthma controls.

## Data Availability

The original contributions presented in the study are included in the article/Supplementary Material, further inquiries can be directed to the corresponding author.
